# ParticleChromo3D+: A Web Server for ParticleChromo3D Algorithm for 3D Chromosome Structure Reconstruction

**DOI:** 10.3390/cimb45030167

**Published:** 2023-03-17

**Authors:** David Vadnais, Oluwatosin Oluwadare

**Affiliations:** Department of Computer Science, University of Colorado, Colorado Springs, CO 80918, USA; dvadnais@uccs.edu

**Keywords:** Hi-C, 3D chromosome structure, chromosome conformation capture, 3D genome

## Abstract

Understanding the three-dimensional (3D) structure of chromatin is invaluable for researching how it functions. One way to gather this information is the chromosome conformation capture (3C) technique and its follow-up technique Hi-C. Here, we present ParticleChromo3D+, a containerized web-based genome structure reconstruction server/tool that provides researchers with a portable and accurate tool for analyses. Additionally, ParticleChromo3D+ provides a more user-friendly way to access its capabilities via a graphical user interface (GUI). ParticleChromo3D+ can save time for researchers by increasing the accessibility of genome reconstruction, easing usage pain points, and offloading computational processing/installation time.

## 1. Introduction

DNA replication, gene regulation, and gene expression are just some of the areas that are affected by chromatin’s three-dimensional (3D) structure [1]. Traditionally, studying chromatin was done via microscopy tools, such as fluorescence in situ hybridization (FISH). FISH has been effective in showing that chromosomes organize in distant 3D territories [2]. Live-cell fluorescence microscopy has even been able to provide insight into the dynamic properties of living cell chromosomes [3]. Chromosome conformation capture (3C) was designed to allow for easier study of these 3D structures. 3C caused a paradigm shift in the way chromatins are studied because it often outperforms FISH [1,4,5]. 3C works through a biochemical procedure that binds and cuts areas of a genome and then measures the frequency at which two sections are bound together [4,6]. Each cut section is known as a chromatin bin, and each bin has a length that is specific to the number of base pairs in the bin [4,6]. Since the advent of 3C, many derivative technologies have been designed, such as Hi-C [7], chromosome conformation capture-on-chip (4C) [8], chromosome conformation capture carbon copy (5C) [9], tethered conformation capture (TCC) [10], and chromatin interaction analysis by paired-end tag sequencing (ChIA-PET) [11]. In particular, Hi-C provides genome-wide chromatin information frequency (IF) data formatted as a contact matrix [7]. Hi-C uses the next-generation sequencing concepts of parallel sequencing and high-throughput sequencing to analyze read-pair interactions on an all-versus-all basis. This means that all of the genome’s read pairs can be sequenced at once [7]. In order to generate the IF data using Hi-C, the data must be pre-processed using data mapping and quality control [12,13]. This involves the process of mapping or aligning the read pairs to a reference genome using read-pair alignment algorithms, such as the Burrows–Wheeler aligner (BWA) [14] or the Bowtie 2 [15]. Following this process is a filtering of the alignment for quality control and, finally, a conversion of the read count data, based on the depth of the resolution, into contact matrix data or maps, which can be plain text, such as a two-dimensional (symmetric) or sparse (three-column) matrix, a binary .hic format [16], or the .cool/.mcool [17] file formats.

The 3D structure reconstruction field is currently focused on developing accurate and robust algorithms to predict the 3D chromosome and genome structure from the IF data produced from in situ Hi-C. In order for a researcher to interpret these Hi-C data, they often pre-process the data into the desired state of 3D positional data using one of a variety of tools [18,19,20,21,22,23,24,25,26,27,28,29,30]. Oluwadare O. et al. wrote a review paper about this in 2019, and, generally, 3D chromosome structure inference tools are based on either distance, contact, or probabilistic methodologies [31]. Distance methods work by first converting the IF data into relative distances and then using optimization techniques to optimize the structure into following the relative distances as closely as possible. Distance-based tools mostly differ from each other according to how the tools perform IF-to-distance conversion and which algorithm is used to optimize the result [31]. Contact methods use IF data directly to perform modeling [31,32]. Finally, probabilistic models measure the probabilistic measure that the chromatin bins will have contact [33]. These methods generally handle noise better than other methods, but they can be more time consuming [31].

In 2022, we published a paper in which we designed and tested a tool for chromosome reconstruction named ParticleChromo3D [18]. ParticleChromo3D is a distance-based tool that reconstructs 3D genome structures using particle swarm optimization (PSO) as its optimization algorithm. PSO is described in detail in our Section 2, but, in general, it is an optimization approach that uses many different particles to explore different configurations [34]. This exploration is completed by having the particles interact/iterate and move towards a local best and global best configuration simultaneously [34]. PSO can become stuck during local best optimizations and may not always find the global best solution [34]. During this, ParticleChromo3D was compared to ChromSDE [21], Chromosome3D [22], 3DMax [23], ShRec3D [24], LorDG [25], HSA [26], MOGEN [27], GEM [28], and PASTIS [29], and it performed well [18]. After creating ParticleChromo3D, we were left with some regrets in that it is not very portable, some knowledge of command line tools is needed to operate it, and it lacks usability and quality-of-life features. We created ParticleChromo3D+ as a containerized web-based implementation of ParticleChromo3D, designed to make it both easier to use externally and highly portable for offline usage.

## 2. Materials and Methods

The web server allows easy access to our three-dimensional (3D) structure optimizer algorithm without having to install Python and the required script dependencies. The algorithm developed in our previous work utilizes a particle swarm optimization (PSO) algorithm to minimize the distance between chromatin bins with the goal of using the interaction frequency to create expected Euclidean distances and then incrementally changing the spatial positioning data to minimize the 3D structure’s expected distance from its current distance [18]. We built the server by running a Docker container that hosts our representational state transfer (REST)-based back-end API and front-end GUI.

### 2.1. Particle Swarm Optimization and ParticleChromo3D

PSO was designed by Kennedy J. and Eberhart R. based on the flight patterns of birds [34]. PSO works by creating a set of particles (in our case, a matrix of chromatin bin locations in [x, y, z]) and then having each particle adjust its position based on a velocity that is a combination of the particle’s history and the best swarm position [18,35]. In general, the position and velocity functions are shown below in Equations (1) and (2), respectively [36].

(1) $$P_{n+1}=P_{n}+V_{n}$$

(2) $$V_{n+1}=w\times V_{n}+c_{1}\times R_{1}\times \left(\Psi_{n}-P_{n}\right)+c_{2}\times R_{2}\times \left(G_{n}-P_{n}\right)$$

where

- $P_{n}$ and $P_{n+1}$ are the position at a time stamp *n* and the position at the next time stamp n+1, respectively.
- $V_{n}$ and $V_{n+1}$ are the velocity at a time stamp *n* and the velocity at the next time stamp n+1, respectively.
- *w* is used to create inertia for the velocity. This helps reduce or increase the contribution of the individual particle’s past velocity.
- $c_{1}$ and $c_{2}$ are the local and global weights, respectively. These weights are used to tell each particle if it should prioritize its information or the swarm information.
- $R_{1}$ and $R_{2}$ are randomized values that increase the breadth of the geometries that the particles explore.
- $\Phi_{n}$ contains the position of the chromatin bin for the optimum structure that the individual particle has found. Each particle has its own structure made up of each chromatin bin’s position.
- $G_{n}$ contains the position of the chromatin bin for the optimum structure that the swarm has found.

**Figure 1:**
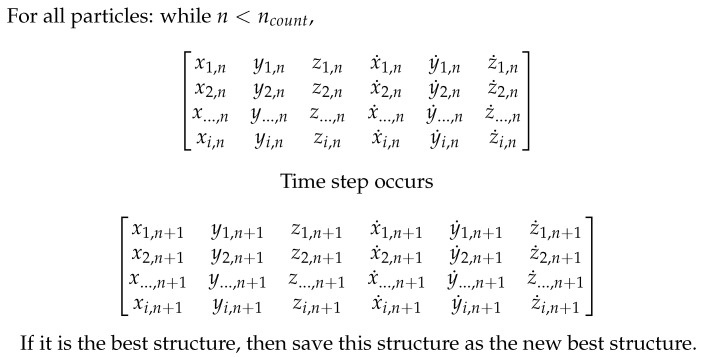
ParticleChromo3D [18] Process Diagram.

Specifically, in ParticleChromo3D (Figure 1), each particle runs one of these update equations on every cell of a matrix at each iteration [18]. This matrix contains continuous numeric data with six features representing the 3D position and velocity of each chromatin bin instance [18]. Each particle’s structure is then compared (see Section 2.2 for details) at every time step to see if it is better than the current best-found structure at all time steps and all particles. If it is the best time structure, it will be saved as the new best structure, and the process will continue until a stop condition is reached.

### 2.2. Scoring Intermittent 3D Structures

In order to know which particle contains the global best structure, we need to be able to rank them. We perform this by comparing the Euclidean distances with a user-selected loss function. We provide the root mean squared error (RMSE) [31], the mean squared error (MSE) [37], the sum of squared errors (SSE) [38], and the Huber loss [39] as options from a drop-down menu. The four loss functions, the RMSE, MSE, SSE, and Huber Loss, are defined below in Equations (7), (4), (5), and (6), respectively.

(3) $$RMSE=\sqrt{\frac{1}{n_{count}}\times \sum {\left(d_{i}-D_{i}\right)}^{2}}$$

(4) $$MSE=\frac{1}{n_{count}}\times \sum {\left(d_{i}-D_{i}\right)}^{2}$$

(5) $$SSE=\frac{\sum \left(d_{i}-D_{i}\right)}{n_{count}}$$

(6) $$HuberLoss=\left\{\begin{array}{cc} \frac{1}{2}\times {\left(d_{i}-D_{i}\right)}^{2} & \mathrm{if}\;(d_{i}-D_{i})\leq \alpha \\ \alpha \times (|d_{i}-D_{i}|-\frac{1}{2}\times \alpha ) & \mathrm{if}\;x=0 \end{array}\right.$$

where:

- $d_{i}$ is the distance between two chromatin bins found by our particle’s structure.
- $D_{i}$ is the expected distance between two chromatin bins based on the IF data.
- $n_{count}$ is the total number of chromatin bins.
- α is a positive real number for alternating between the top and bottom loss functions. We set α to 0.5.

The equation to find $D_{i}$ is [12,31]:

(7) $$D_{i}=\frac{1}{IF_{i,j}^{\beta }}$$

where:

- $D_{i}$ is the expected distance between two chromatin bins based on the IF data.
- $IF_{i,j}$ is the information frequency between two chromatin bins *i* and *j*.
- β is a conversion factor.

### 2.3. Scoring Final 3D Structures

When ParticleChromo3D+ is used, the results will be returned via email as a PDB file, the input parameters, the best optimization’s resulting Spearman correlation coefficient (SCC), and the best Pearson correlation coefficient (PCC). Both the SCC and PCC are defined below [31].

(8) $$PCC=\frac{\sum (\left(d_{i}-\bar{d}\right)\times \left(D_{i}-\bar{D}\right))}{\sqrt{\sum {\left(d_{i}-\bar{d}\right)}^{2}}\times \sqrt{\sum {\left(D_{i}-\bar{D}\right)}^{2}}}$$

where

- $d_{i}$ and $D_{i}$ are individual distances.
- $\bar{d}$ and $\bar{D}$ are sample means of the distances.

(9) $$SCC=\frac{\sum \left(x_{i}-\bar{x}\right)\times \left(y_{i}-\bar{y}\right)}{\sqrt{\sum {\left(x_{i}-\bar{x}\right)}^{2}}\times \sqrt{\sum {\left(y_{i}-\bar{y}\right)}^{2}}}$$

where

- $x_{i}$ and $y_{i}$ are the individual distances $d_{i}$ and $D_{i}$ converted into ranked variables.
- $\bar{x}$ and $\bar{y}$ are sample means of the ranked distances.

### 2.4. Containerization

Containerization is a lightweight competitor to traditional virtual machines (VMs). Containers compete with VMs, reducing the load on the hypervisor [40]. Both containers and VMs, when implemented well, offer isolation, manageability, consolidation, and reliability [41]. Containers also offer better memory efficiency, short cloning times, and numerous continuous integration/continuous delivery (CI/CD) improvements over VMs when lightweight containers are used [42]. We chose to use containerization because we wanted our web server to be as lightweight and portable as possible. To try to gain the advantages of lightweight containers, we based our image on the Apache Tomcat official image sourced from Docker Hub. Our implementation ends up having an image size of roughly one gigabyte and was based on the containerization tool Docker.

### 2.5. Representational State Transfer

**Figure 2:**
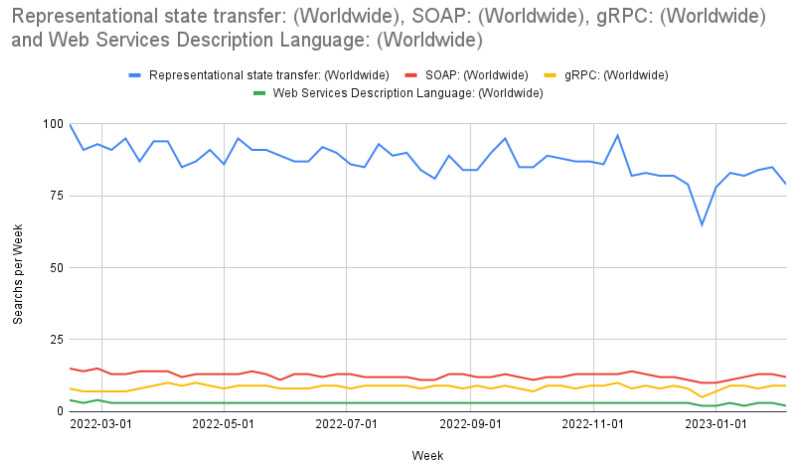
Comparison of REST, SOAP, gRPC, and WSDL searches worldwide.

REST APIs are a mainstay in web-based development [43]. Alternatives to REST APIs include the simple object access protocol (SOAP), web service description language (WSDL), and gRPC (gRPC remote procedure calls) [43,44]. REST APIs assist in offloading storage and computation to cloud computers often through JavaScript object notation (JSON) or hypertext markup language (HTML) International Conference on Web Engineering. We chose REST for our API due to its widespread use, as shown in Figure 2. Figure 2 shows Google Trends’ search count from 13 February 2022 to 5 February 2023 and was sourced from Google Trends on 10 February 2023. An additional reason that we use REST over a non-HTML API is that our data volume does not currently seem to need an implementation such as gRPC, and these implementations would preclude the user from using non-HTML tools, such as Postman.

## 3. Results

ParticleChromo3D+ can be utilized in two ways. The first is to access our web server at http://particlechromo3d.online/ (accessed on 31 January 2023) using a web browser or through back-end services. For web browsers, we tested Firefox and Google Chrome. The second is to host a Docker server and run an instance of the ParticleChromo3D+ image as a container or pod.

### 3.1. Usage

Whether our server or a local container was used, the user can now either upload an IF matrix or use one of the example files therein. This can be done through the front-end GUI or a back-end REST interface via the command line. These implementations default to ports 8080 and 5001, respectively. The GUI is written using HTML and CSS. The REST interface is written using the Python Flask web framework, and it wraps a Python-based script generated for previous research [18].

#### 3.1.1. Front-End Access

**Figure 3:**
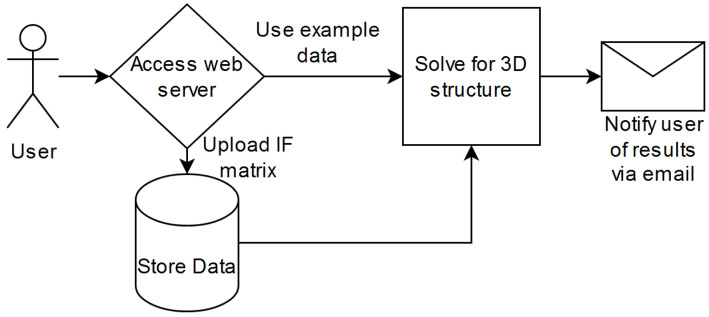
Standard expected use case.

Upon submission of a job to the web server, it will run the particle swarm optimization-based algorithm developed in earlier research through the following process (Figure 3): First, the user can tweak the run parameters. We provide access to the parameters of swarm size, maximum iterations, change threshold, initialization value range, loss function, output file name, and email recipient upon job completion. We provided the default parameters of 15, 30,000, 0.000001, 1.0, and the root mean squared error based on past research. The user must provide a valid email address to begin processing data. Second, the user can choose the IF data they want to process. Third, the data are then sent to the back end and evaluated upon the user pressing the submit button. Finally, the results are emailed to the user and maintained online at a provided download URL.

**Figure 4:**
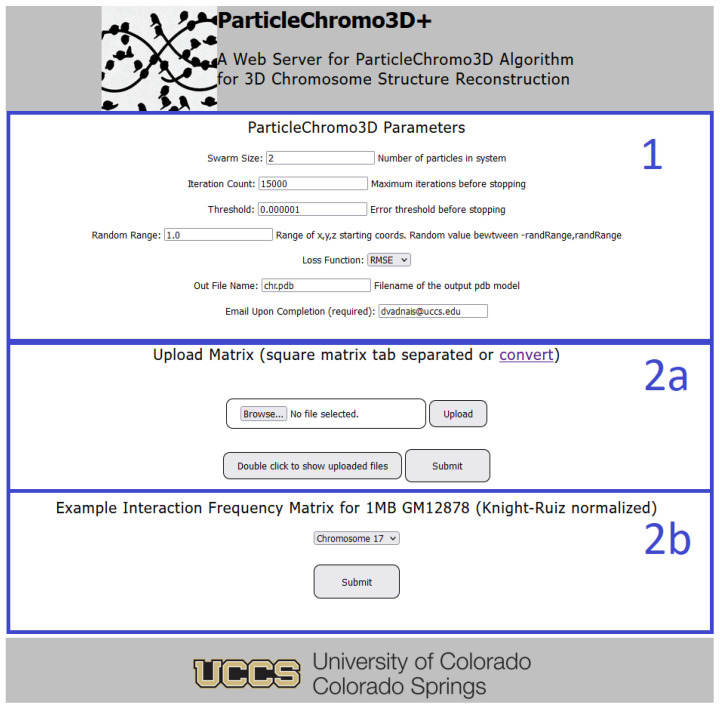
Graphical user interface screenshot for the ParticleChromo3D+ webserver.

The GUI is broken into three parts (Figure 4). First, the optimization parameters are available to be changed. This is labeled as section 1 in Figure 4. Here the user can change or use the provided default values for the Swarm Size, iteration count, error threshold, the random range for the creation of the initial or stating x, y, z coordinate of the 3D chromosome structure, the loss function, or the output file name. The only required field from the user is the provision of their email address to receive the output results. Once the desired optimization parameters have been selected, the user can either upload an IF matrix to process (Figure 4 section 2a) or use the provided 1 mb Hi-C contact map file for the GM12878 cell Hi-C dataset from Rao et al. [45] (Figure 4 section 2b).

#### 3.1.2. Back-End API

The back end can be reached at port 5001 by default. The minimal expected use case is to hit the upload and process endpoints. An sample bash script with examples of many helpful curl commands is provided on GitHub. First, a POST request must be made to the upload endpoint with the desired IF matrix (Table 1). Then, a GET request must be made to the process endpoint with all of its parameters defined (Table 2).

Additionally, we provide access to three more endpoints named uploaded, download, and convert. The uploaded endpoint allows the user to retrieve an HTML formatted list of all of the available IF files for processing. An expected use case for this endpoint is verifying that the desired IF file has been successfully uploaded (accessed on 31 January 2023).
http://biomlearn.uccs.edu:5001/uploaded
The download endpoint allows the user to download the contents of a known name PDB file.
biomlearn.uccs.edu:5001/download?ofname=${filename}
Lastly, the convert endpoint allows the user to convert 3xN matrices to square matrices so long as they are uploaded in a tab-separated values format. This endpoint can be used to format the IF data for use with ParticleChromo3D+ or any other square-matrix-based solution.
biomlearn.uccs.edu:5001/convert?filename=${filename}
Examples of accessing all endpoints via curl can be found at in our GitHub repository under help/exampleCurlScript.bash.

### 3.2. Consistency

**Figure 5:**
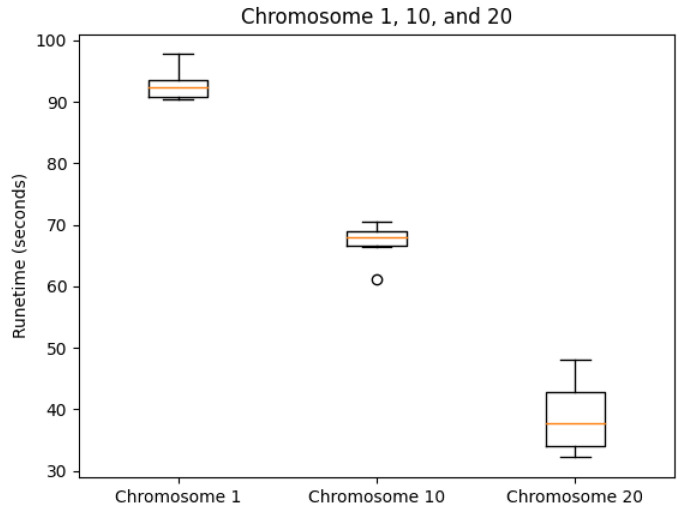
Runtime box plots by chromosome.

**Figure 6:**
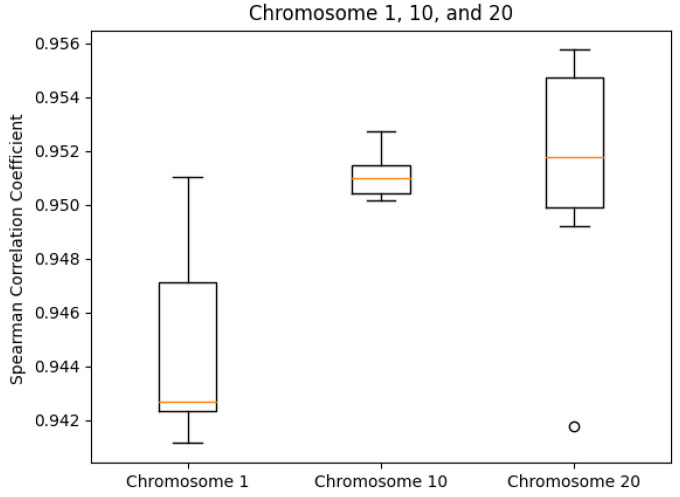
SCC box plots by chromosome.

**Figure 7:**
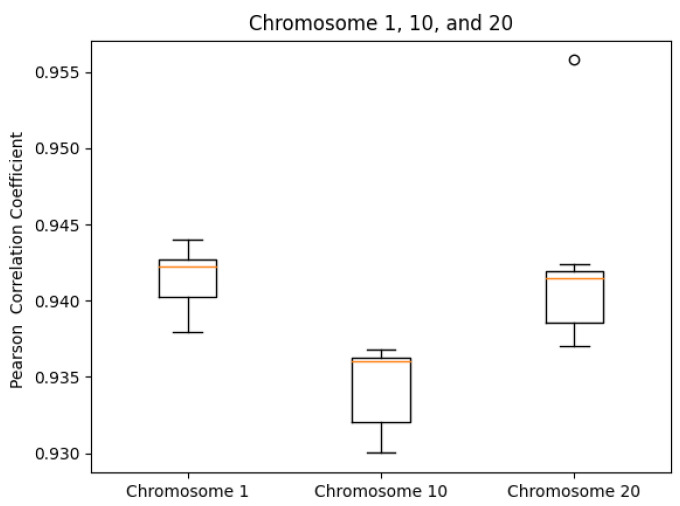
PCC box plots by chromosome.

We tested our server on the GM12878 cell Hi-C dataset, GEO Accession number GSE63525 [45]. The normalized contact matrix was downloaded from the GSDB database with GSDB ID: OO7429SF [46]. We timed our web server’s runtime on Chromosomes 1, 10, and 20 ten times each. While doing this, we recorded the runtime, the best Pearson correlation coefficient, and the best Spearman correlation coefficient from processing start time to the time of email send off completion. The reason that we did not use the time until email received is because the results ended up varying too greatly due to external factors. As expected, the runtime did increase with chromosome size (Figure 5). The maximum runtime was for Chromosome 1 at 97.729 s. The SCC values had ranges of [0.0099, 0.0026, 0.0140] for Chromosomes 1, 10, and 20, respectively. In the same order, the ranges of the PCC values were [0.0060, 0.0068, 0.0188]. Both the SCC and PCC values remained consistent run after run (Figure 6 and Figure 7).

### 3.3. Starting/Extending a Local ParticleChromo3D+ Server

A user may desire to run a local instance of ParticleChromo3D+. Some reasons a user may desire a local instance are for data confidentiality (our research team will never see locally stored data) or offline development environments. If the user needs to run locally, we provide a parameterized way for them to run an instance of ParticleChromo3D+ without worrying about dependencies/builds through GitHub workflows and containerization. Additionally, the user may want to extend our container. A user may want to extend this in order to modify our algorithm or modify our process. By using a standard and open container base, we make it easier to extend our build directly in the Dockerfile or through the FROM notation. We developed ParticleChromo3D+ on a Windows 10 operating system and were able to seamlessly deploy the image on our Linux servers. This supports our goal of portability for the container.

#### 3.3.1. Installation

Our Docker images can be found at https://github.com/OluwadareLab/ParticleChromo3D_Plus/actions (accessed on 31 January 2023) by selecting the desired build and then clicking on the file named particlechromo3d_image.tar.gz under the artifacts section. This image is built by GitHub workflows automatically. Now, the user must load the image with:
 docker image load -i particlechromo3d_image.tar.gz
 docker tag \${IMAGE_ID} particlechromo3d:latest
Once the image has been loaded, it can be run ephemerally or persistently with the instructions on our GitHub homepage. The basic command to run our image is:
docker run -d \
  -p 5001:5001 -p 8080:8080 \
  -e SERVICE_EMAIL=${YOUR_SVC_EMAIL} \
  -e HOSTNAME_BE=${YOUR_URL} \
  -e SERVICE_EMAIL_KEY=${KEY} \
  particlechromo3d:latest
where:

-d runs the container detached.
-p [external:internal] sets the ports.
-e SERVICE_EMAIL is the email address that will be used by the server.
-e HOSTNAME_BE is the DNS name of the server.
-e SERVICE_EMAIL_KEY is the password to the service email.

#### 3.3.2. Extending the Image

If the user wants to extend the image, they can create a new Docker file and use FROM and our image name/tag, or they can build the image and extend our services. To build the image, download or clone our Git repository and then path a Docker CLI into the top-level directory where the Dockerfile is located. In this directory, the user will build the image by running:
 docker build -t particlechromo3D:latest.
If the user wants to make code changes, they should complete this before building the image.

## 4. Discussion

The ParticleChromo3D+ web server provides a GUI and API that give it additional use cases beyond traditional CLI tools. The GUI/API provide ease-of-use for users, and the containerization adds a portable way to convert IF data into 3D chromosome structures. Additionally, the API allows users to procedurally script calls to our server to generate the 3D structures. Through both the API and the front-end web server, researchers can offload the computational load from their servers and place it on ours. Researchers can instantaneously begin processing IF files without having to spend time setting up the algorithm environment. This is achieved while still being comparable to other algorithms in terms of the 3D chromosome structure’s accuracy. ParticleChromo3D+ also has the advantages of offloading work to our servers and removing the need to install required languages and libraries.

## 5. Conclusions

In this work, we present ParticleChromo3D+, a user-friendly web server at http://particlechromo3d.online/ (accessed on 31 January 2023) for predicting 3D chromosome structures from user-uploaded IF data using a particle swarm optimization algorithm [18]. ParticleChromo3D+ reduces the setup time, increases accessibility/usability, and offloads the computational load from research environments. We believe ParticleChromo3D+ is a valuable tool for accelerating genetic research through increasing the accessibility to 3D structure prediction.

## Figures and Tables

**Table 1:** POST http://biomlearn.uccs.edu:5001/upload, (accessed on 31 January 2023).

Parameter	Type	Description
file	Text File	This is your IF matrix

**Table 2:** GET http://biomlearn.uccs.edu:5001/process, (accessed on 31 January 2023).

Parameter	Type	Description
ifname	String	Name of the target IF matrix text file.
ss	Integer	Number of swarms desired.
itt	Integer	Iteration Count. This is an exit condition for the optimization script that defines the maximum iterations before stopping.
threshold	Double	This is an exit condition for the optimization script that defines the threshold of the error minimum before stopping.
randRange	Double	Range of values between which the (x, y, z) initial position can be randomly assigned for each chromatin bin.
lf	Integer [0, 3]	Bit mask for choosing the desired loss function.
outfile	String	Allows the user to tag the out put PDB file’s filename.
email	String	Defines the email address to be messaged upon job completion.

## Data Availability

All real Hi-C data files are available from the GSDB database (accession number(s) OO7429SF). The generated models, all datasets used for all performed analyses, and the source code for ParticleChromo3D are available at https://github.com/OluwadareLab/ParticleChromo3D_Plus, (accessed on 31 January 2023).

## Abbreviations

The following abbreviations are used in this manuscript:

CLI	Command Line Interface
etc.	et cetera
FISH	fluorescence in situ hybridization
gRPC	gRPC Remote Procedure Calls
HTML	Hypertext Markup Language
i.e.	id est
IF	Interaction Frequency
JSON	JavaScript Object Notation
MSE	Mean Squared Error
PCC	Pearson Correlation Coefficient
REST	REpresentational State Transfer
RMSE	Root Mean Squared Error
SCC	Spearman Correlation Coefficient
SOAP	Simple Object Access Protocol
SSE	Sum of Squared Errors
VMs	virtual machines
WSDL	Web Service Description Language
3C	Chromosome Conformation Capture
